# Severe Refractory Vasoplegic Shock Syndrome after OPCABG Successfully Treated with Hydroxycobalamin: A Case Report and Review of the Literature

**DOI:** 10.3390/jcm13010169

**Published:** 2023-12-28

**Authors:** Beatrice Bacchi, Francesco Cabrucci, Bruno Chiarello, Aleksander Dokollari, Massimo Bonacchi

**Affiliations:** 1 Cardiac Surgery F.U., Experimental and Clinical Medicine Department, University of Florence, 50134 Firenze, Italy; beatricebacc@gmail.com (B.B.); francesco.cabrucci.6@gmail.com (F.C.); brunochiarello92@gmail.com (B.C.); 2 Cardiac Surgery Department, St. Boniface Hospital, University of Manitoba, Winnipeg, MB R2H 2A6, Canada; aleksanderdokollari2@gmail.com

**Keywords:** vasoplegic shock syndrome, off-pump CABG, hydroxycobalamin

## Abstract

Background: Vasoplegic shock syndrome (VSS) after an off-pump coronary artery bypass graft (OPCABG) is an extremely rare condition. Inotropic support is usually the first-line therapy, though it can precipitate several complications or be ineffective. We report the first case of severe refractory VSS after OPCABG successfully treated with hydroxycobalamin. Methods: A 77-year-old gentleman underwent OPCABG for three vessels coronary artery disease. Preoperative LV ejection fraction was 28%, and the patient before surgery started sacubitril/valsartan titrated, then, at the highest dose. Surgery was uneventful and, by the end of the procedure, TEE showed improved biventricular contractility. Results: The patient was transferred to the ICU without inotropic support, but soon developed hypotension. TEE ruled out pericardial tamponade and confirmed fair contractility. Norepinephrine was titrated to a medium-high dose, vasopressin was started and a Swan-Ganz catheter was placed. SVR was 480 dyn·s·cm^−5^. Despite aggressive pharmacologic treatment (including methylprednisolone and methylene blue), no improvements were noticed. Ten grams of hydroxycobalamin were administered. One hour later, hemodynamic status re-assessment showed SVR > 800 dyn·s·cm^−5^. Afterward, vasopressors were gradually reduced. Conclusions: Our case demonstrated the importance of adequate early treatment in VSS after OPCABG. This case report shows, for the first time, that hydroxycobalamin was effectively used to restore homeostasis.

## 1. Introduction

Vasoplegic shock syndrome (VSS) is a complex condition arising from a huge imbalance between the loss of systemic vasculature resistance and unresponsiveness to endogen/exogen vasopressor molecules despite a preserved or augmented cardiac output [1]. Although there is still no consensus regarding its definition, VSS is frequently described as exhibiting a cardiac index of 2.2 L/min/m^2^, either normal or elevated, accompanied by a difficulty in maintaining a mean arterial pressure (MAP) of 60 mmHg due to a systemic vascular resistance (SVR) of less than 800 dynes•s/cm^5^, despite receiving high doses of vasopressors and inotropes (equivalent to 0.5 mg/kg/min of norepinephrine) [2]. However, these criteria are relatively non-specific and also found in other disease states such as sepsis, hepatic failure and adrenal insufficiency, among others, with the distinction being the etiology of the shock.

VSS has been also considered a distributive form of circulatory shock developing intraoperatively during a cardiopulmonary bypass (CPB), after weaning from CPB or postoperatively, in the first 24 h, in the intensive care unit (ICU) CPB [3]. VSS, in fact, has been reported in up to 40% of CPB and accounted for less than 5% of all circulatory shock [1]. Refractory VSS carries a mortality rate as high as 25% [4]. 

CPB can be a strong precipitant for VSS and in particular CPB strategies, such as the duration, the employed oxygenator and drain types, priming, myocardial protection, drugs administered during CPB (heparin/protamine) and, most importantly, perfusion pressure. Recirculated blood intraoperatively is also associated with increased inflammatory mediators. 

VSS incidence is higher in patients with preoperative use of angiotensin-converting enzyme inhibitor (ACE-I), calcium channel blockers, amiodarone, heparin, diabetes mellitus or reduced cardiac function (left ventricular ejection fraction < 35%), warmer core temperatures while on bypass, reperfusion injury and long CPB run [5]. 

Although VSS has been extensively studied and described as a post-CPB syndrome, a big question mark remains to explain and treat some reported cases of VSS after off-pump cardiovascular procedures. 

Our study reports the first case of VSS refractory to standard medical treatment occurring after an off-pump cardiac surgery revascularization procedure and successfully managed with hydrossycobalamin infusion. 

## 2. Relevant Sections

### 2.1. Case Report Description

A 77-year-old male with a history of untreated hypertension, dyslipidemia, diabetes mellitus type 1 and major depressive disorder underwent a pre-anesthesia check-up for inguinal hernia surgery. An electrocardiogram revealed a new left bundle branch block. Subsequently, the echocardiogram showed a severely reduced left ventricular ejection fraction (LVEF) of 26%, end-diastolic diameters of 76 mm and end-diastolic volume of 224 mL, with inferior basolateral wall, lateral wall and apex akinesia. The left atrium was also dilated (area 25 cm^2^) and there was mild mitral and tricuspid regurgitation (PAPS 52 mmHg). Therefore, the patient underwent an elective coronary angiogram that revealed 95% stenosis of the proximal left anterior descending artery (LAD), sub-occlusive stenosis of the left circumflex and obtuse marginal (OM) branches and chronic total occlusion of the right coronary artery (RCA). Despite these findings, he had only shortness of breath (class II NYHA). The patient was scheduled for an elective off-pump coronary artery bypass grafting (OPCABG). Before the surgery, the patient was referred to the heart failure outpatient department for optimization of pharmacological therapy, and, starting two weeks before surgery, sacubitril/valsartan was titrated, and then administered at the highest dose. Preoperative, in the operating room, an intra-aortic balloon pump (IABP) was placed. The patient underwent OPCABG and three grafts were performed. The patient received a skeletonized left internal mammary artery (LIMA) to LAD in situ, a skeletonized right internal mammary artery (RIMA) to OM in situ through transverse sinus, and a reverse saphenous vein graft to the posterior descending artery. Surgery was uneventful and, by the end of the procedure, transesophageal echocardiography (TEE) showed improved biventricular contractility.

The patient was transferred to the intensive care unit with IABP and without inotropic support. After about an hour, the patient developed hypotension, tachycardia, and oliguria, initially addressed with fluid management, and a low-dose vasopressor, norepinephrine, was started; however, both treatments failed to improve the hemodynamic condition. TEE ruled out any pericardial tamponade or pleural collections and confirmed fair contractility. A chest X-ray on postoperative day 0 showed no pleuro-parenchymal alterations or pneumothorax. Norepinephrine was titrated to a medium-high dose, vasopressin was started and a Swan-Ganz catheter was placed. SVR was 480 dyn·s·cm^−5^ and the cardiac index was >8.3 L/min/m^2^. A broad spectrum antibiotic was started suspecting sepsis despite no evidence of infection. Despite aggressive pharmacologic treatment and optimization of mechanical ventilation, after 6 h, no improvements in the mean arterial pressure (MAP < 50 mmHg) were noticed and SVR was 500 dyn·s·cm^−5^. Blood lactate levels were continuously rising (peaked up 13 mmol/L), and oliguria was also present. All vasopressors were titrated to the medium-high dose, trying to avoid and limit inotropic-related adverse consequences. Methylprednisolone 200 mg bolus was injected and then repeated at regular intervals daily for one week. An infusion of methylene blue was also started unsuccessfully. 

Being all common conditions that can lead to severe hypotension after cardiac surgery was ruled out, VSS was suspected and appeared as the most reasonable diagnosis. Due to the hemodynamic status of the patient, mechanical circulatory support would not have added any benefit to the clinical condition of the patient and might have deteriorated the SIRS. 

**Figure 1:**
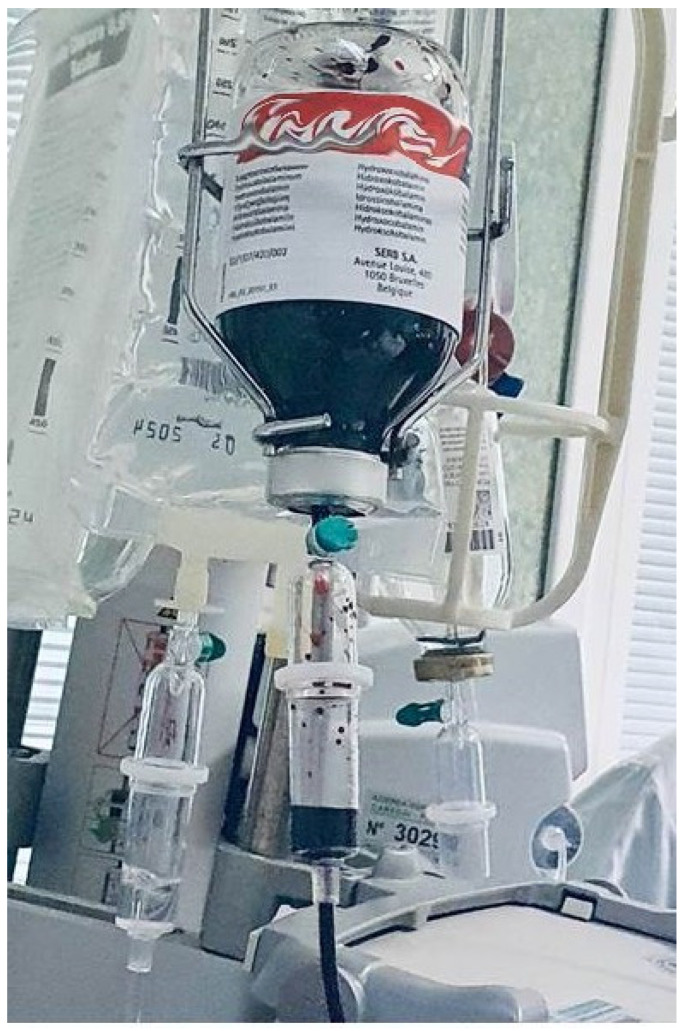
Infusion of hydroxycobalamin.

Ten grams of hydroxycobalamin (labeled used for cyanide poisoning) (Figure 1), was administered as an intravenous infusion by a central line. After about 30 min, MAP started rising and reached 60–65 mmHg. One hour later, MAP was improving, and hemodynamic status re-assessment showed SVR > 800 dyn·s·cm^−5^ and a cardiac index of 4.5 L/min/m^2^. In the following hours, the SBP reached levels above 90 mmHg and SVR was 1100 dyn·s·cm^−5^. Vasopressors were gradually reduced and blood lactate levels decreased. Of note, the chest X-ray on postoperative day 1 described increased congestion of the small circulation and the appearance of bibasal pleural effusion.

As a result of the VSS and its treatment, the patient developed chromaturia, prolonged mechanical ventilation, and percutaneous tracheostomy. The patient was discharged to a rehabilitation facility on postoperative day 26. At the 3-month follow-up, the patient had fully recovered and returned to a normal life. Due to persistent reduced LVEF, an implantable cardioverter-defibrillator (ICD) was implanted. The latest echocardiogram showed positive remodeling of the left ventricle and no residual regional wall motion abnormalities. No major adverse cardiac and cerebrovascular events (MACCE) were reported at the 1-year follow-up.

### 2.2. Literature Review

This review was carried out in accordance with the Preferred Reporting Items for Systematic Reviews and Meta-Analyses (PRISMA) guidelines. The following databases were searched for studies meeting our inclusion criteria and published by 1 September 2023: PubMed/MEDLINE and Google Scholar. We searched for the following terms: [“vasoplegic shock”] and/or [“vasoplegic syndrome after off-pump CABG”]. 

Studies were excluded if any of the following criteria were met: (1) vasoplegic shock or syndrome after cardiopulmonary bypass; (2) not published in English language; (3) not published in a peer-reviewed journal; and (4) was a conference abstract.

After the removal of duplicates and articles not in English language, there were four papers presenting cases of VSS occurring in off-pump CABG (Table 1). 

## 3. Discussion

Our literature review on VSS after off-pump cardiac procedures, Table 1, confirmed the need for an alternative effective medical treatment to inotropic support in case of severe refractory VSS, even in OPCABG. Even though early diagnosis can be challenging, understanding VSS pathophysiology is crucial to promptly start adequate therapy. 

A delayed diagnosis, especially in the initial phase, could result in an unpredictable prognosis. Aggressive treatment is usually required, yet this process needs to be continuously tailored to the hemodynamic status of the patient. 

The pathophysiology of VSS is multifactorial, and its underlying processes are often likened to septic shock [6]. The combination of exposure of blood to the foreign surfaces of CPB and surgical trauma triggers a systemic inflammatory response, considered a causative factor in the development of vasoplegia. During surgery, tissue injury and hypoperfusion can activate immune cells (i.e., macrophages and monocytes) leading to the release of pro-inflammatory cytokines such as TNF-α, interleukin-1β (IL-1β) and IL-6. These cytokines decrease the responsiveness of vascular smooth muscle cells to vasoconstrictors, causing vasodilation and decreased SVR. They also contribute to activating inducible nitric oxide synthase (iNOS) enzymes, promoting the release of large volumes of NO. iNOS enzymes are, in fact, stimulated by inflammatory cytokines and can lead to significantly higher levels of NO compared to constitutive endothelial NOS. The release of NO prevents smooth muscle calcium influx and hyperpolarizing smooth muscle cells through the activation of adenosine triphosphate (ATP)-sensitive potassium channels, leading to systemic vasodilatation [7]. The activation of the complement system can also contribute to vasoplegia by releasing vasoactive peptides, such as bradykinin and C5a, leading to vasodilation and increased vascular permeability [8]. Moreover, CPB not only increases NO production and ATP depletion, but it also enhances vascular smooth muscle acidemia, resulting in decreased myosin phosphorylation and vasodilation. Simultaneously, neuro-hypophyseal deposits from endogenous vasopressin are depleted rapidly, adding to the vasodilation effect, and creating vasoplegia [9].

In the present case, VSS developed after OPCABG, despite two of the main pathways of VSS pathophysiology (systemic inflammatory response (SIRS) and cellular hyperpolarization through the inactivation of Ca^2+^ voltage-gated channels) [1] being less exhibited. Therefore, other possible etiologies in the absence of extracorporeal circulation should be considered. 

In the case of off-pump procedures, the activation of pro-inflammatory cytokines and iNOs appears to be initiated by heparin-protamine administration with all the consequences of the development of SIRS and multi-organ failure due to hypotension refractory to catecholamines [5]. 

In addition to this mechanism, it is possible that preoperative chronic congestive heart failure with low EF and therapy with sacubitril/valsartan, started at a high dose just before surgery, could have precipitated the systemic inflammatory response related to surgery and VSS. LVEF < 35–40%, in fact, is an independent predictor of vasoplegia after cardiac surgery with CPB. A pre-existing, increased inflammatory profile along with the compensatory chronic activation of the sympathetic nervous system and the renin–angiotensin–aldosterone system may be responsible for the development of postoperative vasoplegia in patients with heart failure [10]. 

To our knowledge, to date, this is the first case reporting the possible association of sacubitril/valsartan and VSS in OPCABG. Previous data regarding the use of sacubitril/valsartan and VSS after cardiac surgery are controversial [11], although some case reports have described profound vasoplegia after CPB [12]. Haider et al. [11] reported the preoperative use of sacubitril-valsartan in patients undergoing heart transplantation or left ventricular assist device (LVAD) surgery and found that the angiotensin receptor neprilysin inhibitor was not significantly associated with the development of vasoplegic syndrome. Similar results were also described by Domínguez et al., in their multicentric study, in which the incidence of vasoplegic syndrome associated with the preoperative use of sacubitril-valsartan in a cohort of 96 patients undergoing heart transplant was 15.6% [13]. Conversely, Almufleh et al. [12] described in their case report the association of postoperative vasoplegia with preoperative use of sacubitril-valsartan in a patient undergoing a heart transplant. 

Nowadays, current management recommendations in cardiac surgery encourage stopping sacubitril-valsartan before the procedure. However, considering that the half-life of the drug and its metabolites reaches 18 h [14] and that not all cardiac surgery procedures can be scheduled, it is very likely that some patients undergoing treatment with this drug can be under its effect at the time of the surgical intervention. 

The possible involvement of the sacubitril-valsartan in the mechanisms of vasoplegic syndrome is also supported by the fact that the renin–angiotensin–aldosterone system (RAS) is one of the three different systems (together with the sympathetic and vasopressinergic system) usually involved in the maintenance of blood pressure. RAS antagonists such as angiotensin-converting enzyme (ACE) inhibitors and angiotensin receptor blockers (ARB) block, in fact, the RAS response to hypotension. Therefore, a complete suppression of the RAS due to this treatment might play a crucial role in VSS [15]. In addition, considering that anesthetic drugs reduce the influence of the sympathetic system on cardiovascular tone, under general anesthesia, an increased reliance on RAS and the vasopressinergic system can maintain blood pressure. 

Gomes et al. [16]. described a case series of four patients who underwent VSS after OPCABG (total incidence 0.4% in 5 years). All patients were treated with high doses of norepinephrine, presented postoperative complications related to the VSS and one patient died. Interestingly, they found soaring levels of tumor necrosis factor α (TNF-α) suggesting that this cytokine is a likely mediator of this syndrome even in off-pump procedures. 

Norepinephrine is usually the first-line therapy in case of hypotension associated with vasodilatory shock since it increases vascular and venous resistance, cardiac preload, and inotropy, and reduces vascular capacitance. Norepinephrine has vascular effects both on the arterial and on the venous side, with an increase of both arterial and venous pressure. Nevertheless, in some patients, the blood pressure constantly rises, whereas cardiac output might not increase. Therefore, restoring blood pressure with norepinephrine infusion may not always be associated with an improvement in tissue perfusion [17]. In particular, the Vasoplegic Shock after Cardiac Surgery (VANCS) trial evaluated the role of norepinephrine specifically in vasoplegia treatment versus vasopressin as a first-line therapy in patients recovering from cardiac surgery [18]. In this trial, the primary short-term endpoint of a composite of mortality or severe complications was higher in the norepinephrine group. However, norepinephrine did not differ from vasopressin concerning mortality, and the differences in this endpoint were driven by more arrhythmia and acute kidney injury specifically. Higher doses of any catecholamine, in fact, may be associated with immunosuppression, an increase in myocardial oxygen demand, interference with cellular energy metabolism, oxidative stress, arrhythmias and risk of necrosis secondary to severe peripheral vasoconstriction [19]. 

As an alternative, vasopressin has gained popularity in restoring vascular tone and increasing clinical outcomes [18] as shown by Raja et al. [20]. In this case report, however, before obtaining negative microbiology results, the patient was treated as a septic shock-like case, delaying the proper diagnosis. Moreover, as suggested in the article, despite an off-pump procedure significantly decreasing the systemic inflammatory response syndrome usually developed after a cardiac surgery intervention, some cases of refractory vasoplegia developed after OPCABG. Therefore, the generation of proinflammatory mediators due to surgical stress, the use of re-sterilized disposable devices and protamine, the transfusion of blood products or the occurrence of endotoxemia secondary to repeated episodes of hypotension throughout the surgery because of mobilization and displacement of the heart could precipitate the systemic inflammatory response and vasoplegic syndrome [20]. In addition, vasopressin may also modulate the production of NO as well as potentiate the adrenergic response to stress [21]. Finally, the use of vasopressin is particularly attractive in vasoplegia considering the depletion of this molecule occurring during CPB. Several factors can contribute to this reduction, but most likely the neurohumoral effects of elevated cardiac filling pressures preoperatively, elevations in atrial natriuretic peptide (ANP) or autonomic dysregulation are the most predominant. 

Other vasoactive drugs used include phenylephrine (alpha-1 receptor agonist), ascorbic acid, thiamine and angiotensin II (angiotensin II receptor type 1 agonist, causing vasoconstriction and upregulating endogenous vasopressin secretion) [22]. Angiotensin II in the setting of post-CPB vasoplegia is particularly attractive, as extra-corporeal circulation would be expected to bypass pulmonary circulation and thereby limit exposure of angiotensin I to ACE. In the ATHOS-3 study, patients with VSS who were receiving more than 0.2 μg.kg^−1^.min^−1^ of norepinephrine or the equivalent dose of another vasopressor for more than 6 h were assigned to receive infusions of either angiotensin II or placebo. A significant increase after 3 h of infusion was reached by more patients in the angiotensin II group than in the placebo group (*p* < 0.001). However, in this study, only 16 of the 344 patients underwent cardiac surgery [23].

The use of corticosteroids in the setting of VSS is associated with the mechanisms of these agents in restoring vascular responsiveness to vasopressors, likely through a non-genomic inhibition of the arachidonic acid cascade and a genomic inhibition of the nuclear translocation of the NF-κB transcription factor [24], together with the inhibition of the synthesis of iNOS and COX2 [25]. 

In particular, a combination of high doses of ascorbic acid, 50 mg hydrocortisone every 6 h and thiamine 200 mg/12 h is recommended as an adjuvant therapy in addition to the other vasopressors. This combination is known to cause a rapid and significant reduction in vasopressor requirement, rapidly restores the MAP and improves mortality [26]. 

Dopamine can be also added since it increases systemic vascular resistance and inotropism, though it has been associated with an increased risk of arrhythmias compared with other catecholamines [27]. 

Currently, no data are supporting one non-catecholamine therapy over the others. Therefore, if a single infusion of a single drug cannot achieve the target pressure, a second drug with a different mechanism of action should be used. Therefore, initial therapy usually involves volume expansion and administration of norepinephrine [18]. Second-line vasopressors might include other catecholamines, like phenylephrine, epinephrine and dopamine, while other agents that can be used are arginine vasopressin, methylene blue, ascorbic acid, hydroxocobalamin, corticosteroids and angiotensin II. However, to note, significant inconsistency exists between countries and clinical centers in the management of vasodilatory shock [28] and yet, the mortality rate remains high, mainly due to inadequate cellular oxygen utilization and multi-organ failure, especially acute kidney injury (AKI).

Even though the treatment of VSS is challenging, the aim is to address every single step in the pathophysiologic cascade of VSS to restore homeostasis. Inotropic agents are just a supportive therapy that can lead to resistance or even severe toxic effects at high doses [22]. Therefore, focusing the treatment on hypotension using an uncontrolled escalade of vasopressor infusion can be not only useless but also deleterious leading to a catastrophic vicious circle of malperfusion. It has been demonstrated that vasopressors have a non-responding threshold at the highest dose [29,30]. Once the splanchnic and peripheral malperfusion are established, these processes will further deteriorate the SIRS and the energetic status of the cells resulting in the complete loss of SVR. 

Dysregulation of NO release, production or signaling plays a key role in the systemic vasodilation of vasoplegic and septic shock. Therefore, targeted therapies such as methylene blue and hydroxocobalamin acting to interrupt this pathway might be promptly considered. Methylene blue is a water-soluble dye that inhibits NO synthase and guanylate cyclase to reverse vasodilation caused by excessive NO signaling. Given its effectiveness as both a preventive and rescue therapy, inhibiting both constitutive and inducible nitric oxide synthase, as well as guanylate cyclase, it is considered the treatment of choice. However, methylene blue can precipitate serotonin syndrome if associated with serotonergic antidepressants since it is a potent inhibitor of monoamine oxidase [31]. Our patient, although they had a history of major depressive syndrome, was not under more medical treatment for 20 years. Therefore, methylene blue was administered excluding the risk of serotoninergic syndrome. Unlike methylene blue, hydroxocobalamin has not been associated with the serotonergic pathway or serotonin syndrome. 

Hydroxycobalamin for catecholamine-resistant VSS during CPB was first described by Roderique et al. [32]. In this case report, the author described the occurrence of vasoplegic syndrome while on CPB in a patient undergoing CABG and valve surgery. The refractory hypotension was initially treated with phenylephrine and norepinephrine but with minimal response. Therefore, hydroxycobalamin was administered with benefit. 

To the best of our knowledge, our report is the first one describing the use of hydroxycobalamin treatment for VSS after OPCAB. 

Hydroxycobalamin is usually used to counteract the excess of nitric oxide (NO) released into the bloodstream by cyanide poisoning. Therefore, it is considered a scavenging molecule for large amounts of NO released by inducible nitric oxide synthase [32]. Hydroxycobalamin also enhances the clearance of vasodilators such as hydrogen sulfides and endothelium-hyperpolarizing factors, restoring, thus, the balance between vasodilator and vasoconstrictor molecules [33]. This re-established equilibrium generally can bring a recovery in the efficacy of vasopressors on vessels’ smooth muscles. Given its off-label use in treating vasoplegia, the dosage of this drug is based on its primary indication of cyanide poisoning—5–10 g IV infusion over 15 min. However, the literature reports extending the duration of infusion of the same 5 g dose (median 6 h, range 1–10 h), to significantly and gradually reduce the vasopressor requirements throughout the infusion, which appeared to be sustained after cessation [34]. Hydroxocobalamin side effects include chromaturia, nausea, erythema, nephrolithiasis, lymphocytopenia and infusion site reactions. Of note, our patient developed only the first complication listed. Hydroxycobalamin could be used as the last resource in an attempt to treat severe refractory VSS leading to an unfavorable prognosis or might be used as a vasopressor-sparing agent at an earlier stage trying to avoid the vasopressor-induced malperfusion. 

In our case, methylene blue therapy was attempted first, but the patient successfully responded when hydroxocobalamin was added. This phenomenon can suggest either a synergistic phenomenon of hydroxocobalamin with methylene blue or the presence of an alternative pathway. Many case reports, in fact, report the successful combination of hydroxocobalamin with methylene blue [4,35] compared to the use of methylene blue alone [36]. On the other hand, as previously described, hydroxycobalamin is a significant molecule restoring the balance between vasodilator and vasoconstrictor molecules counteracting not only the excess of NO but also promoting the clearance of some other vasodilator factors. 

**Figure 2:**
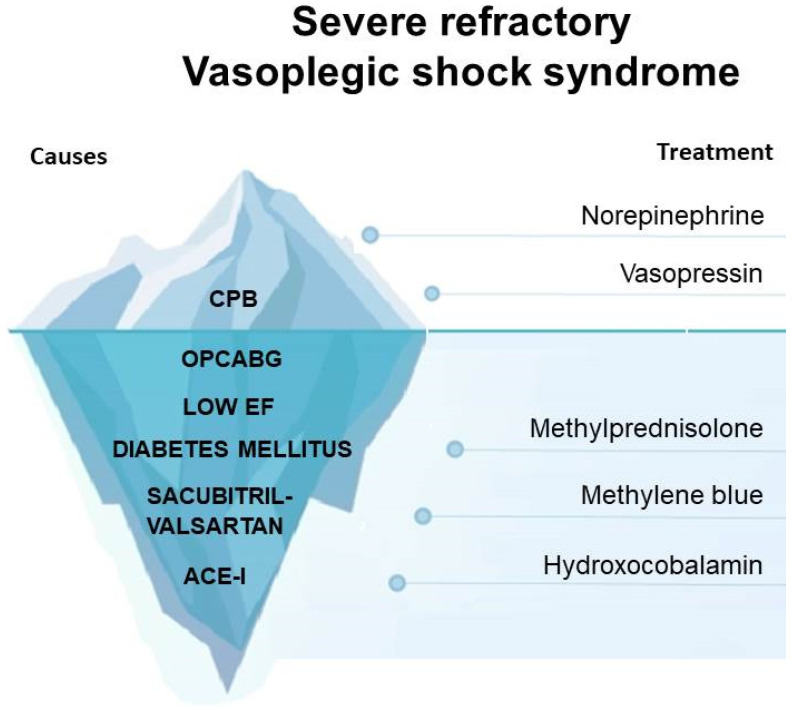
CPB: Cardiopulmonary bypass; OPCABG: off-pump coronary artery bypass grafting; EF: ejection fraction; ACE-I: angiotensin-converting enzyme inhibitors.

Although hydroxycobalamin is a promising molecule, its response can be unpredictable [20], and considering its high cost [37], its usage should be balanced in a fair allocation of resources. Acknowledging that our case has the limitation of a single case report and that further investigations are needed to evaluate the real strength of hydroxycobalamin in OPCABG, we strongly believe that the management of this case could be a useful tool for the physician facing this complex life-threatening complication and that our findings may be a starting point for future studies(Figure 2).

**Table 1:** Vasoplegic shock syndrome after OPCABG.

Study Author	Year of the Study	Type of the Study	Patients Included	Treatment
Sun et al. [3]	2008	comparative	10	-
Gomes et al. [16]	2003	retrospective	4	Norepinephrine
Raja et al. [20]	2004	case report	1	Norepinephrine and vasopressin
Vaidyanathan et al.	2017	case report	1	Norepinephrine and vasopressin

## 4. Conclusions

Our case demonstrates the importance of early recognition of severe refractory VSS in OPCABG, since SIRS after reperfusion injury of a severe ischemic heart, even without CPB, can be extremely large. Moreover, with the increasing clinical use of sacubitril-valsartan in heart failure patients, accurate preoperative planning should be performed, when feasible, to reduce the possible effects of this drug on the postoperative course. Finally, hydroxycobalamin can play a crucial role in restoring homeostasis in such a challenging situation. The usage of this molecule in a similar setting could pave the way for future research.

## Data Availability

The data that support the findings of this study are available upon reasonable request to the corresponding author.

## References

[1] Ltaief Z., Ben-Hamouda N., Rancati V., Gunga Z., Marcucci C., Kirsch M., Liaudet L. (2022). Vasoplegic Syndrome after Cardiopulmonary Bypass in Cardiovascular Surgery: Pathophysiology and Management in Critical Care. J. Clin. Med..

[2] Ortoleva J.P., Cobey F.C. (2019). A systematic approach to the treatment of vasoplegia based on recent advances in pharmacotherapy. J. Cardiothorac. Vasc. Anesth..

[3] Gomes W.J., Carvalho A.C., Palma J.H., Gonçalves I., Buffolo E. (1994). Vasoplegic syndrome: A new dilemma. J. Thorac. Cardiovasc. Surg..

[4] Levin M.A., Lin H.M., Castillo J.G., Adams D.H., Reich D.L., Fischer G.W. (2009). Early on-cardiopulmonary bypass hypotension and other factors associated with vasoplegic syndrome. Circulation.

[5] Sun X., Zhang L., Hill P.C., Lowery R., Lee A.T., Molyneaux R.E., Corso P.J., Boyce S.W. (2008). Is incidence of postoperative vasoplegic syndrome different between off-pump and on-pump coronary artery bypass grafting surgery?. Eur. J. Cardiothorac. Surg..

[6] Burgdorff A.M., Bucher M., Schumann J. (2018). Vasoplegia in patients with sepsis and septic shock: Pathways and mechanisms. J. Int. Med. Res..

[7] Busse L.W., Barker N., Petersen C. (2020). Vasoplegic syndrome following cardiothoracic surgery-review of pathophysiology and update of treatment options. Crit. Care.

[8] Ni Choileain N., Redmond H.P. (2006). Cell response to surgery. Ni Choileain, N.; Redmond HP. Arch. Surg..

[9] Muhammad R., Dharmadjati B.B., Mulia E.P.B., Rachmi D.A. (2022). Vasoplegia: Mechanism and Management Following Cardiopulmonary Bypass. Eurasian J. Med..

[10] Papazisi O., Bruggemans E.F., Berendsen R.R., Hugo J.D.V., Lindeman J.H.N., Beeres S.L.M.A., Arbous M.S., van den Hout W.B., Mertens B.J.A., Ince C. (2022). Prevention of vasoplegia with CytoSorb in heart failure patients undergoing cardiac surgery (CytoSorb-HF trial): Protocol for a randomised controlled trial. BMJ Open.

[11] Haider L., Hugon-Vallet E., Constantin J.P., Riad Z., Sebbag L., Mewton N. (2021). ARNI Pre-Operative Use and Vasoplegic Syndrome in Patients Undergoing Heart Transplantation or Left Ventricular Assist Device Surgery. Med. Sci..

[12] Almufleh A., Mielniczuk L.M., Zinoviev R., Moeller A., Davies R.A., Stadnick E., Chan V., Chih S. (2018). Profound Vasoplegia During Sacubitril/Valsartan Treatment After Heart Transplantation. Can. J. Cardiol..

[13] Domínguez J.M., García-Romero E., Pàmies J., Mirabet S., González-Costello J., Spitaleri G., Perez-Villa F., Farrero M. (2020). Incidence of vasoplegic syndrome after cardiac transplantation in patients treated with sacubitril/valsartan. Clin. Transplant..

[14] Kobalava Z., Kotovskaya Y., Averkov O., Pavlikova E., Moiseev V., Albrecht D., Chandra P., Ayalasomayajula S., Prescott M.F., Pal P. (2016). Pharmacodynamic and Pharmacokinetic Profiles of Sacubitril/Valsartan (LCZ696) in Patients with Heart Failure and Reduced Ejection Fraction. Cardiovasc. Ther..

[15] Ozal E., Kuralay E., Yildirim V., Kilic S., Bolcal C., Kücükarslan N., Günay C., Demirkilic U., Tatar H. (2005). Preoperative methylene blue administration in patients at high risk for vasoplegic syndrome during cardiac surgery. Ann. Thorac. Surg..

[16] Gomes W.J., Erlichman M.R., Batista-Filho M.L., Knobel M., Almeida D.R., Carvalho A.C., Catani R., Buffolo E. (2003). Vasoplegic syndrome after off-pump coronary artery bypass surgery. Eur. J. Cardiothorac. Surg..

[17] Andrei S., Bar S., Nguyen M., Bouhemad B., Guinot P.G. (2023). Effect of norepinephrine on the vascular waterfall and tissue perfusion in vasoplegic hypotensive patients: A prospective, observational, applied physiology study in cardiac surgery. Intensive Care Med. Exp..

[18] Hajjar L.A., Vincent J.L., Barbosa Gomes Galas F.R., Rhodes A., Landoni G., Osawa E.A., Melo R.R., Sundin M.R., Grande S.M., Gaiotto F.A. (2017). Vasopressin versus Norepinephrine in Patients with Vasoplegic Shock after Cardiac Surgery: The VANCS Randomized Controlled Trial. Anesthesiology.

[19] Hartmann C., Radermacher P., Wepler M., Nussbaum B. (2017). Non-Hemodynamic Effects of Catecholamines. Shock.

[20] Raja S.G., Dreyfus G.D. (2004). Vasoplegic syndrome after off-pump coronary artery bypass surgery: An unusual complication. Tex. Heart Inst. J..

[21] Omar S., Zedan A., Nugent K. (2015). Cardiac vasoplegia syndrome: Pathophysiology, risk factors and treatment. Am. J. Med. Sci..

[22] Papazisi O., Palmen M., Danser A.H. (2022). The use of angiotensin II for the treatment of post-cardiopulmonary bypass vasoplegia. Cardiovasc. Drugs Ther..

[23] Khanna A., English S.W., Wang X.S., Ham K., Tumlin J., Szerlip H., Busse L.W., Altaweel L., Albertson T.E., Mackey C. (2017). Angiotensin II for the treatment of vasodilatory shock. N. Engl. J. Med..

[24] Bellissant E., Annane D. (2000). Effect of hydrocortisoneon phenylephrine mean arterial pressure dose-response relationship in septic shock. Clin. Pharmacol. Ther..

[25] Bailey J.M., Makheja A.N., Pash J., Verma M. (1988). Corticosteroids suppress cyclooxygenase messenger RNA levels and prostanoid synthesis in cultured vascular cells. Biochem. Biophys. Res. Commun..

[26] Marik E., Khangoora V., Rivera R., Hooper M.H., Catravas J. (2017). Hydrocortisone, vitamin C, and thiamine for the treatment of severe sepsis and septic shock: A retrospective before-after study. Chest.

[27] De Backer D., Biston P., Devriendt J., Madl C., Chochrad D., Aldecoa C., Brasseur A., Defrance P., Gottignies P., Vincent J.L. (2010). Comparison of dopamine and norepinephrine in the treatment of shock. N. Engl. J. Med..

[28] Abril M.K., Khanna A.K., Kroll S., Mc Namara C., Handisides D., Busse L.W. (2019). Regional differences in the treatment of refractory vasodilatory shock using angiotensin II in high output shock (ATHOS-3) data. J. Crit. Care.

[29] Shah R., Wenger R.K., Patel P.A., Davis S., Ha B., Feinman J.W., Patel S., Pulton D., Weiss S.J., Restrepo-Cardenas J. (2020). Severe Vasoplegic Shock During Coronary Artery Bypass Surgery: Therapeutic challenges and Dilemmas in Hemodynamic Rescue. J. Cardiothorac. Vasc. Anesth..

[30] Wieruszewski P.M., Khanna A.K. (2022). Vasopressor Choice and Timing in Vasodilatory Shock. Crit. Care.

[31] Oz M., Lorke D.E., Hasan M., Petroianu G.A. (2011). Cellular and molecular actions of Methylene Blue in the nervous system. Med. Res. Rev..

[32] Roderique J.D., VanDyck K., Holman B., Tang D., Chui B., Spiess B.D. (2014). The use of high-dose hydroxycobalamin for vasoplegic syndrome. Ann. Thorac. Surg..

[33] Charles F.G., Murray L.J., Giordano C., Spiess B.D. (2019). Vitamin B12 for the treatment of vasoplegia in cardiac surgery and liver transplantation: A narrative review of cases and potential biochemical mechanisms. Can. J. Anaesth..

[34] Seelhammer T.G., Plack D., Nei S., Wittwer E., Nelson J., Nabzdyk C.G. (2021). Extended duration infusion of high-dose hydroxocobalamin for vasoplegic syndrome following cardiac surgery. Heart Lung.

[35] Levin R.L., Degrange M.A., Bruno G.F., Del Mazo C.D., Taborda D.J., Griotti J.J., Boullon F.J. (2004). Methylene blue reduces mortality and morbidity in vasoplegic patients after cardiac surgery. Ann. Thorac. Surg..

[36] Shah P., Reynolds P., Pal N. (2018). Hydroxocobalamin for the treatment of cardiac-associated vasoplegia: A case series. Can. J. Anaesth..

[37] Shapeton A.D., Mahmood F., Ortoleva J.P. (2019). Hydroxocobalamin for the Treatment of Vasoplegia: A Review of Current Literature and Considerations for Use. J. Cardiothorac. Vasc. Anesth..

